# Challenges in intradural disc herniation diagnosis and surgery: A case report

**DOI:** 10.1016/j.amsu.2020.08.022

**Published:** 2020-09-09

**Authors:** Muhamad Thohar Arifin, Novita Ikbar K, Surya P. Brilliantika, Yuriz Bakhtiar, Jacob Bunyamin, Zainal Muttaqin

**Affiliations:** Department of Neurosurgery, Faculty of Medicine, Diponegoro University, Semarang, Indonesia

**Keywords:** Intradural herniation, Lumbar herniated disc, Microsurgery

## Abstract

**Introduction:**

Intradural disc herniation (IDH) is a rare complication which comprises 0.27% of all herniated intervertebral discs. We are reporting a case of lumbar intradural disc herniation at the L4-L5 levels highlighting challenges in establishing clinical diagnosis and surgical approach involving a transdural microsurgery approach.

**Presentation of case:**

A 38-year-old gentleman was presented with left radicular low back pain without motoric and autonomic involvement admitted to our neurosurgical service. Spine MRI showed an intradural, extra-axial spinal mass.

**Discussion:**

Lumbar IDH is a rare pathology thus often initially diagnosed as other more common conditions. In our case, the IDH diagnosis was confirmed during surgery as the radiological examination results mimic intradural extra-axial tumor. During surgery, a hard irregular white mass was found shortly after dural incision. Histopathological results showed chondrocytes, fibrotic and necrotic appearances consistent with the diagnosis of disc herniation. Postoperatively, the patient showed improvement and pain alleviation.

**Conclusion:**

We observed the beak sign which is one of the important features of IDH imaging. Surgery-wise, the challenge of dissecting the anterolateral part of the duramater from the annulus fibrosus of the intervertebral disc should be noted by the performing surgeon.

## Highlights

• Intradural disc herniation (IDH) is a rare complication and comprises 0.27% of all herniated disc.
• Radiological findings mimicking for intradural extra-axial spine tumors.
• Challenge in dissecting the duramater of the anterolateral segments is a common problem.
• A median or paramedian dural incision accompanied by a microsurgical technique.
• Better visibility of hernia after a dural incision, avoiding damage to the nervous rootlets.

## 1 Introduction

Intradural disc herniation (IDH) is a rare condition where the nucleus pulposus displaced into the dural sac of intervertebral disc. It comprises 0.27% of all herniated discs. The first case was published by Dandy in 1942, and is commonly located at the lumbar region (92%) [1,2]. It mainly appears at the L4-L5 level (55%), followed by L3-L4 level (16%), L5-S1 level (10%), while the upper lumbar discs were rarely affected. Because of its extradural location, IDH is sometimes misdiagnosed as other spinal abnormalities such as schwannoma, ependymoma or metastasis preoperatively [3]. Hereby, we present a case of lumbar intradural disc herniation at the levels of L4-L5 which was initially diagnosed as an intradural, extra-axial tumor according to the SCARE 2018 guideline [4].

## 2 Presentation of Case

**Fig. 1:**
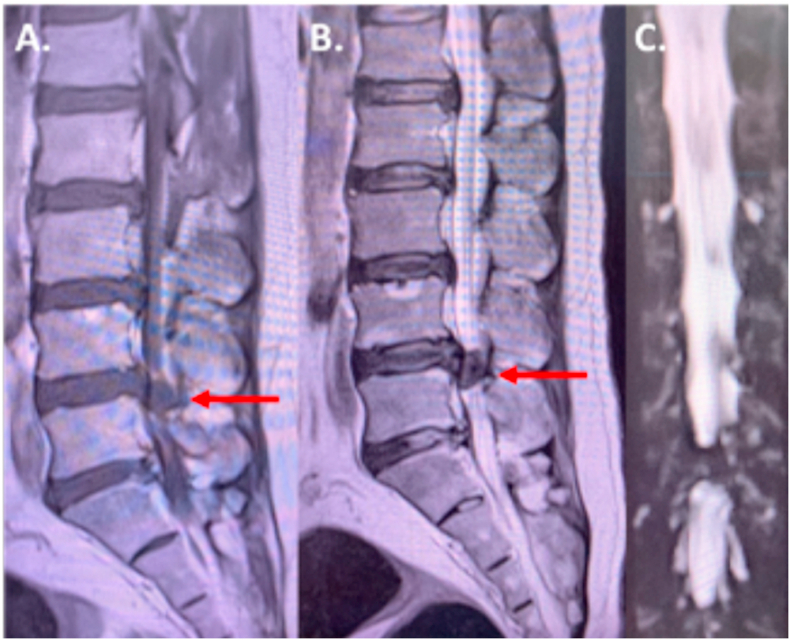
Sagittal section lumbar MRI showed a hypointense mass after gadolinium-injection on T1-weighted image (red arrow) (A) a heterogenous hypointense mass on T2-weighted image (red arrow) (B) and mass-like lesion on the MR myelography (C). (For interpretation of the references to colour in this figure legend, the reader is referred to the Web version of this article.)

**Fig. 2:**
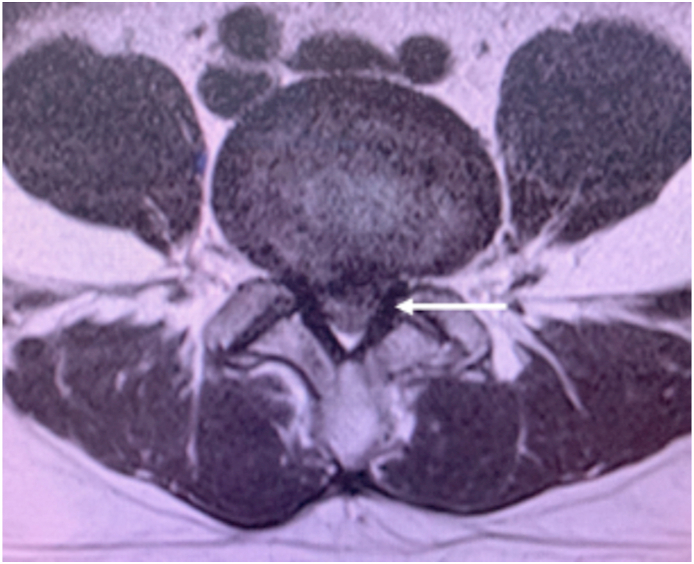
Axial section of L4-L5 level showing the beak-like appearance and eripheral enhancement of the lesion after gadolinium injection (white arrow).

A 38 year-old Asian gentleman was referred to our neurosurgical service due to disabling low back pain radiating down the left lower limb for two years. He denied tingling and burning sensation, weakness, nor bladder and bowel problems. In the medical history, there was no co-existing conditions were found. Clinical examination corresponded with L4-L5 roots involvement with positive SLR test. Past medical, surgical, and familial history were insignificant. The T2-weighted sagittal MRI showed a hypointense heterogenous mass at the left L4-L5 level with a beak-like lesion compressing the spinal cord on the axial section (Fig. 1, Fig. 2). Myelogram showed a complete block of contrast flow on the same level. The initial diagnosis was an intradural, extra-axial spine mass with the possibility of schwannoma, ependymoma or metastasis.

**Fig. 3:**
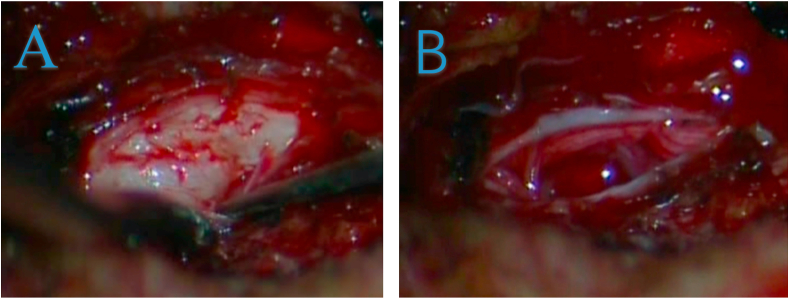
Intraoperative photograph (A) outlining the peripheral displacement of the adherent cauda equine nerve roots (white arrows) by the large intradural disc fragment and (B) post mass-extraction.

**Fig. 4:**
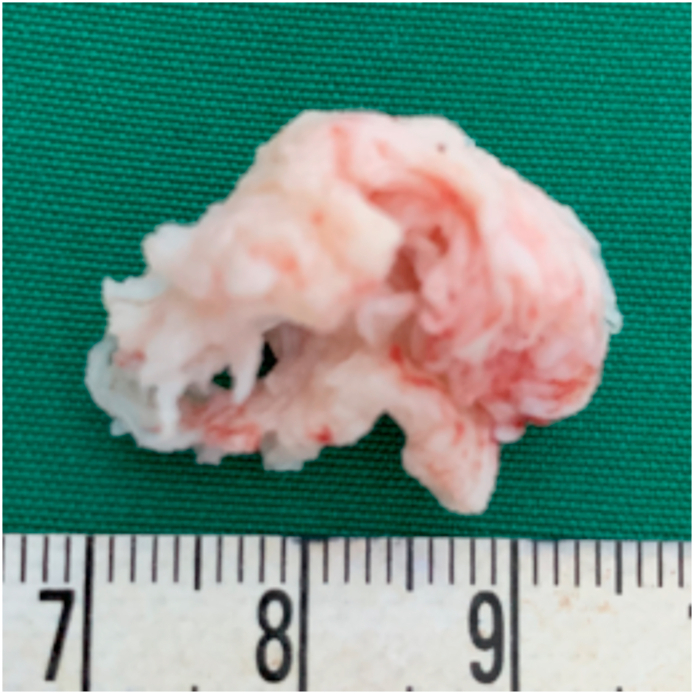
The hard, irregular white mass specimen measured 2 cm × 1.5 cm x 1 cm.

He was scheduled for hemilaminectomy of the left L4 and L5 levels. The surgery was performed by Z.M., M.T.A and Y.Z who were attending neurosurgeons. Surgery was performed in prone, anti-lordotic position under general anesthesia from the left side. Intra-operatively, a hard, irregular white mass was discovered shortly after dural incision and was easily removed without firm adhesion (2 cm × 1.5 cm x 1 cm) (Fig. 3, Fig. 4). Pathological results confirmed of chondrocytes, fibrotic and necrotic appearances, which were consistent with the diagnosis of disc herniation. Postoperatively, the patient experienced a significant relieve of sensory disturbance on his left lower limb and thus discharged home.

## 3 Discussion

Intradural disc herniation is a rare complication which comprises 0.27% of all herniated discs. It is commonly found at the lumbar region, especially the L4-L5 levels. It poses a diagnostic challenge as the radiological findings could mimic intradural and extra-axial spinal tumors such as schwannoma. Therefore, it is important to include IDH as a differential diagnosis for intradural and extra-axial mass. Generally, the predisposing factor is the dense adhesion between the dural sac and posterior longitudinal ligament [5]. In normal condition, this ligament and the duramater are loosely attached, however there are some possible cause of the dense adhesions [6]. Such type of adhesions have been suggested as the etiopathogenesis of herniated lumbar disc [7].Traumatic irritations such as prior surgery and chronic disc herniation may lead to the adhesion [8]. Chronic local inflammation and congenital fusions are also thought to be the possible cause of adhesion [5]. This event might be co-occurring with chronic disc herniation which weakens the duramater furthermore and predispose it to spontaneous intradural rupture. In newborns, IDH was also reported possibly due to idiopathic causes [9,10].

IDH could be difficult to diagnose, however several MRI signs have been described to aid establishing preoperative diagnosis. Following the gadolinum administration, the most common IDH findings is ring enhancement appearance due to peripheral neovascularization and chronic granulation. However, ring enhancement appearance could be witnessed in other spinal tumors such as schwannoma. In T2-weighted sequence, a sharp beak-like appearance or hawk beak shape is an indicator for IDH which is shown in our case. Preoperative crumb disc is another common sign for IDH, which is caused by an irregularly bordered mass. Based on the anatomical attribute, IDH is classified into two types. The type A in which disc herniation into protrudes into the dural sac is often located in the lower cervical and lumbar region. The type B is disc herniation through the dural sheath of nerve roots, thus could only be diagnosed intra-operative. The main treatment of IDH is surgical removal of the herniated disc material. Meanwhile, the duramater and nerve roots require careful attention during surgical exploration.

Challenges in exploring the duramater of the anterolateral segments is a common problem. In previous reports, thick adhesions were present between the disc space and the dura mater at the time of surgery while the dural sac tended to be rough, tense and immobile [8,11,12]. Therefore, microsurgical approach by a median or paramedian dural incision is recommended for better visibility of the hernia to avoid damage to the nervous rootlets. The nerve rootlets should be minimally stimulated to avoid neurological dysfunction. In our case, the patient only reported preoperative radicular pain and had had good recovery with no further neurological problems.

## 4 Conclusion

Lumbar IDH is a rare pathology thus often initially diagnosed as other more common conditions. In our case, the IDH diagnosis was confirmed during surgery as the radiological examination results mimic intradural extra-axial tumor. We observed the beak sign which is one of the important features of IDH imaging. Surgery-wise, the challenge of dissecting the anterolateral part of the duramater from the annulus fibrosus of the intervertebral disc should be noted by the performing surgeon.

## Ethical approval

Ethical approval exempted by our institution.

## Sources of funding

No funding

## Author contribution

1. Muhamad Thohar Arifin: Performed surgery, conception of report, data collection, data analysis, manuscript writing, revision and submission.
2. Novita Ikbar K: data collection, data analysis, manuscript writing and manuscript revision.
3. Surya P Briliantika: data collection, data analysis, manuscript writing and manuscript revision.
4. Yuriz Bakhtiar: performed surgery, conception of report, data collection, data analysis, manuscript writing and manuscript revision
5. Jacob Bunyamin: data analysis, manuscript writing and manuscript revision.
6. Zainal Muttaqin: performed surgery, conception of report, data collection, data analysis, manuscript writing and manuscript revision

## Registration of research studies

Case report not registered.

## Guarantor

Zainal Muttaqin, Profeesor Of Neurosurgery Department Faculty Of Medicine Diponegoro University.

## Consent

Written informed consent was obtained from the patient. Information within the paper has been sufficiently anonymised not to cause harm to the patient or family.

## Provenance and peer review

Not commissioned, externally peer reviewed.

## Declaration of competing interest

No conflicts of interest.
